# High proportions of bacteria and archaea across most biomes remain uncultured

**DOI:** 10.1038/s41396-019-0484-y

**Published:** 2019-08-06

**Authors:** Andrew D. Steen, Alexander Crits-Christoph, Paul Carini, Kristen M. DeAngelis, Noah Fierer, Karen G. Lloyd, J. Cameron Thrash

**Affiliations:** 1 Department of Microbiology, University of Tennessee, Knoxville, TN 37996 USA; 2 Department of Earth and Planetary Sciences, University of Tennessee, Knoxville, TN 37996 USA; 3 Department of Plant and Microbial Biology, University of California, Berkeley, CA USA; 4 Department of Environmental Science, BIO5 Institute, University of Arizona, Tucson, AZ USA; 5 Department of Microbiology, University of Massachusetts, Amherst, MA 01003 USA; 6 Department of Ecology and Evolutionary Biology, Cooperative Institute for Research in Environmental Sciences, University of Colorado, Boulder, CO 80305 USA; 7 Department of Biological Sciences, University of Southern California, Los Angeles, CA 90089 USA

**Keywords:** Biodiversity, Microbial ecology

## Abstract

A recent paper by Martiny argues that “high proportions” of bacteria in diverse Earth environments have been cultured. Here we reanalyze a portion of the data in that paper, and argue that the conclusion is based on several technical errors, most notably a calculation of sequence similarity that does not account for sequence gaps, and the reliance on 16S rRNA gene amplicons that are known to be biased towards cultured organisms. We further argue that the paper is also based on a conceptual error: namely, that sequence similarity cannot be used to infer “culturability” because one cannot infer physiology from 16S rRNA gene sequences. Combined with other recent, more reliable studies, the evidence supports the conclusion that most bacterial and archaeal taxa remain uncultured.

What fraction of bacterial and archaeal cells on Earth have been cultured? This simple question has a complex answer. In 1932, Razumov showed that cultivation-based cell enumerations yield far smaller estimates of cell abundance than microscopic counts [1]. More recently, it has become clear that many microbial taxa are so distantly related to laboratory strains that they belong to entirely new families, orders, or even phyla with no cultured representatives [2, 3]. Most bacterial phyla lack cultured representatives [3]. Quantifying the fraction of cells on Earth that are represented in culture collections remains an important—but essential—analytical and conceptual challenge given that microbial physiology is an emergent property that is not easily inferred from sequence data alone.

In a recent Brief Communication, Martiny’s answer to this question is summarized in the title, “High proportions of bacteria are culturable across major biomes”, and his final sentence, “…many if not most abundant lineages across diverse biomes are already in culture” [4]. Here we argue that these conclusions are based on inadequate and inherently biased datasets and analyses as well as the mistaken logic that an organism is culturable if its 16S rRNA gene sequence is >97% similar to a laboratory culture. Below we highlight existing evidence that the majority of cells in most environments are only distantly related to organisms that have been cultured.

First, Martiny relies on full length, PCR-amplified 16S rRNA gene sequences to assess the abundance of microbial taxa in the environment. PCR primers commonly used to amplify 16S rRNA genes are biased toward certain taxa and completely miss newly discovered lineages, some of which can be identified from metagenomes [5–7].

Because shotgun sequencing of environmental DNA does not rely on sequence-specific PCR amplification prior to sequencing, it is not biased in the same way as amplification-based approaches. Thus, metagenomes are expected to contain 16S rRNA gene sequences in proportions that more closely approximate their absolute abundances in environmental DNA. A recent meta-analysis comparing 16S rRNA gene sequences retrieved from either amplicon or metagenomic datasets showed that commonly used primers targeting the 16S rRNA gene yielded a considerably higher abundance of cultured organisms across most publically available datasets [8]. While the magnitude of the difference between primer-based analyses and metagenomic-based analyses will differ depending on PCR primer selection and the environment in question, PCR-based assessments of culturability considerably and consistently over-represent the fraction of cultured organisms relative to metagenomic-based studies. To estimate the magnitude of this effect, we compared abundances of 16S rRNA gene sequences obtained by PCR amplification to abundances obtained from metagenomic libraries, in each of 14 broad environment types, using data originally reported in Lloyd et al. [8]. Sequence abundance in metagenomic libraries was measured as the median number of reads recruiting to each sequence, as reported by IMG/M [9], which represents the abundance of each 16S rRNA gene sequence in DNA extracted from each sample. We note that the PCR-amplified data and metagenomic data come from different studies, so they represent trends within environments rather than a head-to-head comparison of results from identical samples.

Second, Martiny used the SeqMatch tool provided by the Ribosomal Database Project (RDP) to identify sequence similarity between environmental 16S rRNA gene sequences and isolates [10]. SeqMatch reports a “similarity score” that does not include internal gapped alignment positions and only reports differences between aligned bases. This score is calculated differently from common identity scores between full length sequences, including default scores for programs such as BLAST, USEARCH, CD-HIT, UPARSE, and mothur, which count internal sequence insertions and deletions as biologically valid differences [11]. Because the length of the 16S rRNA genes can vary considerably [12], gaps are important markers of evolutionary distance [6]. The SeqMatch tool also reports sequence identity to the best hit in the RDP alignment, which does not align the most variable regions of the 16S rRNA gene [13], yet these regions are the most likely to contain substitutions between species. In addition, 1–2% of the sequences identified by SeqMatch as the closest isolate match to environmental sequences in the Martiny analysis are misidentified as isolates, and can be traced back to unidentified environmental sequences (Tables S1 and S2). These database errors would have introduced a small additional bias towards overestimating the abundance of cultured sequences. Lastly, the limitation of the analysis to only 100 sequences per sample is also likely to greatly underestimate the numbers of taxa identified from a given sample.

To demonstrate how database and analysis choice can impact results, we reanalyzed all sequences from Jangid et al. [14] and Santelli et al. [15] from the Martiny analysis, along with 48,000 representative OTU sequences obtained by Karst et al. [16] (Fig. 1). The Karst et al. data set was generated using novel methods based on physical concentration, size selection, and both short- and long-read sequencing of small subunit rRNA molecules, which is free from primer bias. We report sequence identities from the *align.seqs* command in the mothur package (counting internal, and not external, sequence gaps as biological differences) to both reported isolates and type strains in the SILVA [17] database (47,917 sequences), and a version of the RDP database filtered to contain only isolates based on RDP’s “isolate filter” (513,426 sequences). We found lower median best hit percent sequence identities than those reported by Martiny, and often much lower percentages of sequences with >97% identity to an isolate in the dataset without primer biases than in the PCR studies. Only 6% of the representative OTU sequences reported by Karst et al. [16] were found to have >97% identity to any isolate in the larger filtered RDP dataset. Methods are described in more detail in the supplemental. Our revised results are closer to what was found for a large database, including nearly all published metagenomes, of which 2–18% of 16S rRNA gene sequences were >96.6% similar to a sequence from a cultured microbe [8]. This cutoff of 96.6% similarity is the upper 95% confidence interval of the median sequence identity within genera [16]. It is also worth noting that another study using the SILVA database and only full-length sequences concluded that “18.9% of bacterial sequences and 6.8% of archaeal sequences have come from isolated organisms”, although there was great variation across phyla [18]. However, in no phylum were sequences from cultured organisms in the majority (Table 1).

**Fig. 1:**
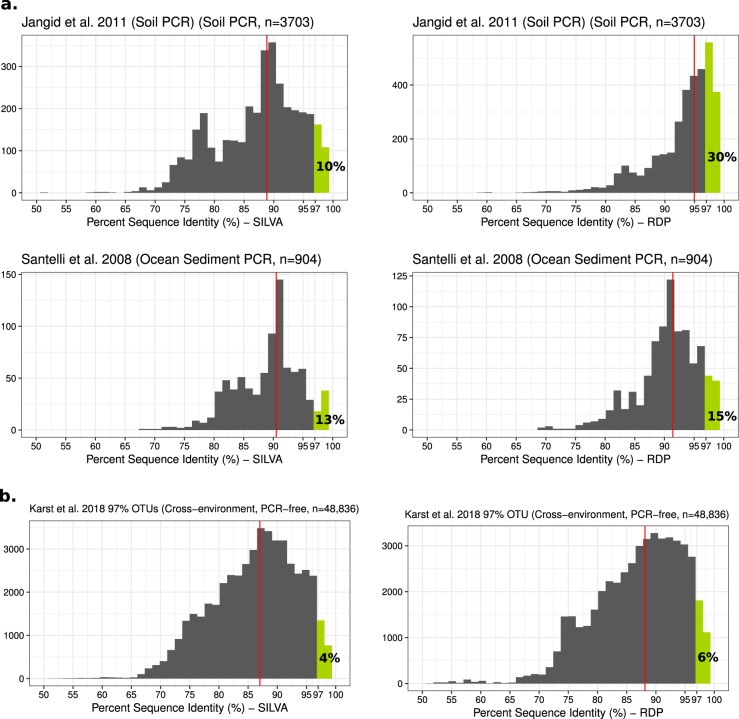
Best hit percent identities of environmental sequences to both isolate 16S rRNA gene sequences in the SILVA database (left column) and a filtered version of the RDP database (right column) from **a** two PCR-based studies analyzed in Martiny 2019, and **b** a large set of dereplicated OTU sequences free from primer bias and from diverse environments [16]. Median best hit percent identities for each dataset are marked with a red line and the percentage of sequences, which had best hits at >97% identity are marked in green

**Table 1:** Enrichment of 16S rRNA gene sequences from organisms at different levels of taxonomic novelty due to PCR amplification, values are calculated as the ratio of the median fraction of 16S rRNA gene sequences in 4743 amplicon-based datasets to the median read depth of 16S rRNA gene sequences in 1504 unamplified metagenomes. (Data from figures 2 and 3 of Lloyd et al. [8])

Enrichment in amplicon libraries vs metagenome-derived sequences	Bacteria median (25th percentile, 75th percentile)	Archaea median (25th percentile, 75th percentile)
Cultured species (≥96.6% 16S rRNA gene similarity)	180% (140, 330%)	270% (180, 430%)
Uncultured genus to class	98% (76, 110%)	70% (55, 120%)
Uncultured phylum	54% (32, 82%)	70% (46, 94%)

Finally, we disagree on the conceptual linkage between “culturability” and 16S rRNA gene sequence similarity to a culture. The 97% 16S rRNA gene sequence similarity cutoff is frequently used to group microbes into species [19, 20], and it is true that microbes within this cutoff often share many traits that are important to culturability [21]. However, a great deal of genomic and phenotypic diversity can exist among strains of the same species [22, 23], with important implications for an organism’s ecological function and the conditions required to culture it [24–27]. Physiological state can also influence culture yields: for instance, variable rates of dormancy can have profound impacts on assessments of “culturability” within a single population [28]. Thus, it is impossible to reliably assess whether an organism is “culturable” based on 16S rRNA gene sequence alone.

We agree with Martiny that the oft-repeated axiom that only 1% of microbial cells in all environments are culturable should be retired. As Martiny clearly lays out, the precise meaning of this statement is often nebulous, and the underlying data are hard to identify. One recent meta-analysis found that a median of 0.5% of cells identified in direct counts could be grown in culture using standard techniques [8], but the variance was large (interquartile range from 0.025 to 4.3%; Fig S1). Individual studies have found culturability yields of 70% or more in environments as diverse as the human gut, surface marine sediments, and lakes in which volcanic ash had recently been deposited [29–31]. Furthermore, the term “culturable” only makes sense in the context of specified culturing conditions, a distinction that Martiny draws in the difference between H1 (that 1% of cells can be cultured) and H3 (that 1% of cells will grow on a standard agar plate). Culturing efforts to characterize microbial communities should rely on diverse methods and media to maximize yield [32] and methodological innovations have opened the door to culturing previously uncultured taxa [33–37].

Thus, we argue that it is impossible to know whether a microbe is culturable until it has been cultured, so the term “unculturable” should be avoided in favor of “uncultured.” Historically, the cultivation and study of microbes in the laboratory has been integral to our understanding of the microbial roles in the world around us, and even in the age of bioinformatics it remains essential. In order to understand the degree to which we have characterized the microbial world, we need to measure the fraction of microbial cells on Earth that share physiologies with cultured cells. Getting this estimate right is an important academic question, but it also has implications for allocation of resources by funding agencies. An overestimate of the degree to which culture collections reflect the Earth’s microbiome would discourage funders from supporting efforts to culture important, uncultured taxa. We hope that future culturing efforts will be successful enough that most bacteria and archaea from most environments will have representation in culture collections. At present, however, the best evidence indicates that this is very far from the case.

## Supplementary information

Table S1

Table S2
