# Supplementary material for: High proportions of bacteria and archaea across most biomes remain uncultured
Source: ISME J. 2019 Aug 6;13(12):3126–30. doi: 10.1038/s41396-019-0484-y (PMC6863901; doi:10.1038/s41396-019-0484-y)
Supplement: Supplementary file 1 — Table S1 [file 41396_2019_484_MOESM1_ESM.pdf]

Env Sequence	Top SeqMatch ‘isolate’ RDP Database Hit (actually environmental sequences)	“Percent Similarity” (not including gaps)
U011088045|EU491325.1	Chloroflexi bacterium SCGC AAA240-F16; HQ675582	0.989
U011088181|EU491189.1	Nitrospina sp. SCGC AAA240-J07; HQ675602	0.952
U011087943|EU491427.1	alpha proteobacterium SCGC AAA007-F15; HQ675463	0.943
U011087977|EU491393.1	Chloroflexi bacterium SCGC AAA240-O15; HQ675645	0.908
U011088182|EU491188.1	Planctomycetes bacterium SCGC AAA166-J20; JF488353	0.886
U011087939|EU491431.1	Planctomycetes bacterium SCGC AAA003-J23; HQ675420	0.893
U011088053|EU491317.1	Chloroflexi bacterium SCGC AAA240-O05; HQ675640	0.905
U011088049|EU491321.1	Chloroflexi bacterium SCGC AAA240-F16; HQ675582	0.984
U011088152|EU491218.1	Nitrospina sp. SCGC AAA240-J07; HQ675602	0.929
U011088223|EU491147.1	gamma proteobacterium SCGC AAA240-I16; HQ675598	0.954
U011088219|EU491151.1	Planctomycetes bacterium SCGC AAA158-E19; JF488487	0.973
U011088144|EU491226.1	Gemmatimonadetes bacterium SCGC AAA240-M10; HQ675622	0.921
U011088211|EU491159.1	Planctomycetes bacterium SCGC AAA001-J07; HQ675380	0.858
U011088270|EU491100.1	gamma proteobacterium SCGC AAA007-K08; HQ675473	0.938
U011088327|EU491043.1	gamma proteobacterium SCGC AAA007-P21; HQ675492	0.954
U011088091|EU491279.1	Planctomycetes bacterium SCGC AAA166-J20; JF488353	0.895
U011088214|EU491156.1	SAR406 cluster bacterium SCGC AAA288-D02; HQ675666	0.933
U011088326|EU491044.1	bacterium SCGC AAA071-D13; JF488657	0.909
U011088030|EU491340.1	Acidobacterium sp. SCGC AAA240-L09; HQ675616	0.975
U011088215|EU491155.1	Nitrospina sp. SCGC AAA240-J07; HQ675602	0.94
U011088119|EU491251.1	Chloroflexi bacterium SCGC AAA240-B13; HQ675545	0.858
