# Supplementary material for: High proportions of bacteria and archaea across most biomes remain uncultured
Source: ISME J. 2019 Aug 6;13(12):3126–30. doi: 10.1038/s41396-019-0484-y (PMC6863901; doi:10.1038/s41396-019-0484-y)
Supplement: Supplementary file 2 — Table S2 [file 41396_2019_484_MOESM2_ESM.pdf]

Env Sequence	Top SeqMatch ‘isolate’ RDP Database Hit (actually environmental sequences)	“Percent Similarity” (not including gaps)
U011088679|EF665718.1	Gemmatimonadetes bacterium SCGC AAA007-O06; HQ675488	0.951
U011088397|EF666001.1	gamma proteobacterium SCGC AAA007-P21; HQ675492	0.949
U011088742|EF665655.1	Verrucomicrobia bacterium SCGC AAA160-P04; JF488614	0.857
U011088783|EF665614.1	bacterium SCGC AAA018-L5; HQ290505	0.929
U011088642|EF665755.1	Bacteroidetes bacterium SCGC AAA204-N13; JF488165	0.978
